# Oscillatory dynamics within fronto-insular-parietal networks integrate thermosensory signals into body ownership

**DOI:** 10.1016/j.isci.2026.117679

**Published:** 2026-09-26

**Authors:** Giulia Mattavelli, Francesco Crottini, Irene Gorrino, Eleonora Arrigoni, Carlotta Maiocchi, Alberto Pisoni, Gabriella Bottini, Gerardo Salvato

**Affiliations:** 1 IUSS Cognitive Neuroscience (ICoN) Center, Scuola Universitaria Superiore IUSS, Piazza della Vittoria 15, 27100 Pavia, Italy; 2 Cognitive Neuroscience Laboratory of Pavia Institute, IRCCS Istituti Clinici Scientifici Maugeri, Via Salvatore Maugeri 10, 27100 Pavia, Italy; 3 Psychology Department, University of Milano-Bicocca, Piazza dell'Ateneo Nuovo 1, 20126 Milan, Italy; 4 Cognitive Neuropsychology Centre, ASST “Grande Ospedale Metropolitano” Niguarda, Piazza Ospedale Maggiore 3, 20162 Milan, Italy; 5 Department of Brain and Behavioral Sciences, University of Pavia, Corso Strada Nuova 65, 27100 Pavia, Italy; 6 Milan Center for Neuroscience – NeuroMi, Piazza dell'Ateneo Nuovo 1, 20126 Milan, Italy

**Keywords:** temperature, body ownership, bodily self-awareness, thermosensory signals

## Abstract

Cutaneous thermosensory signals are linked to survival and homeostatic regulation, yet their relationship with higher order cognitive functions remains poorly understood. Emerging evidence suggests that skin temperature is associated with body ownership—the sense that a body and its parts belong to oneself—but the oscillatory basis of this relationship in healthy individuals is unknown. We combined a multisensory illusion paradigm with thermal imaging and electroencephalography to investigate the oscillatory correlates of skin-temperature changes during experimentally induced changes in body ownership. Selective perturbations in theta/low-alpha oscillatory activity, localized to right premotor regions, tracked a decrease in hand temperature during altered hand ownership. Moreover, the strength of a functional connectivity network spanning bilateral frontoparietal areas, including the insula, was associated with temperature changes coupled with ownership modulation. These findings identify a previously uncharacterized oscillatory signature associated with thermosensory changes during body ownership alteration, linking physiological fluctuations to neural dynamics of self-representation.

## Highlights

• Body ownership modulation induces bilateral reductions in hand temperature
• Theta/low-alpha activity in right frontoparietal regions correlates with cooling
• Frontoinsular-parietal connectivity links thermosensory signals and ownership

## Graphical abstract

**Figure:**
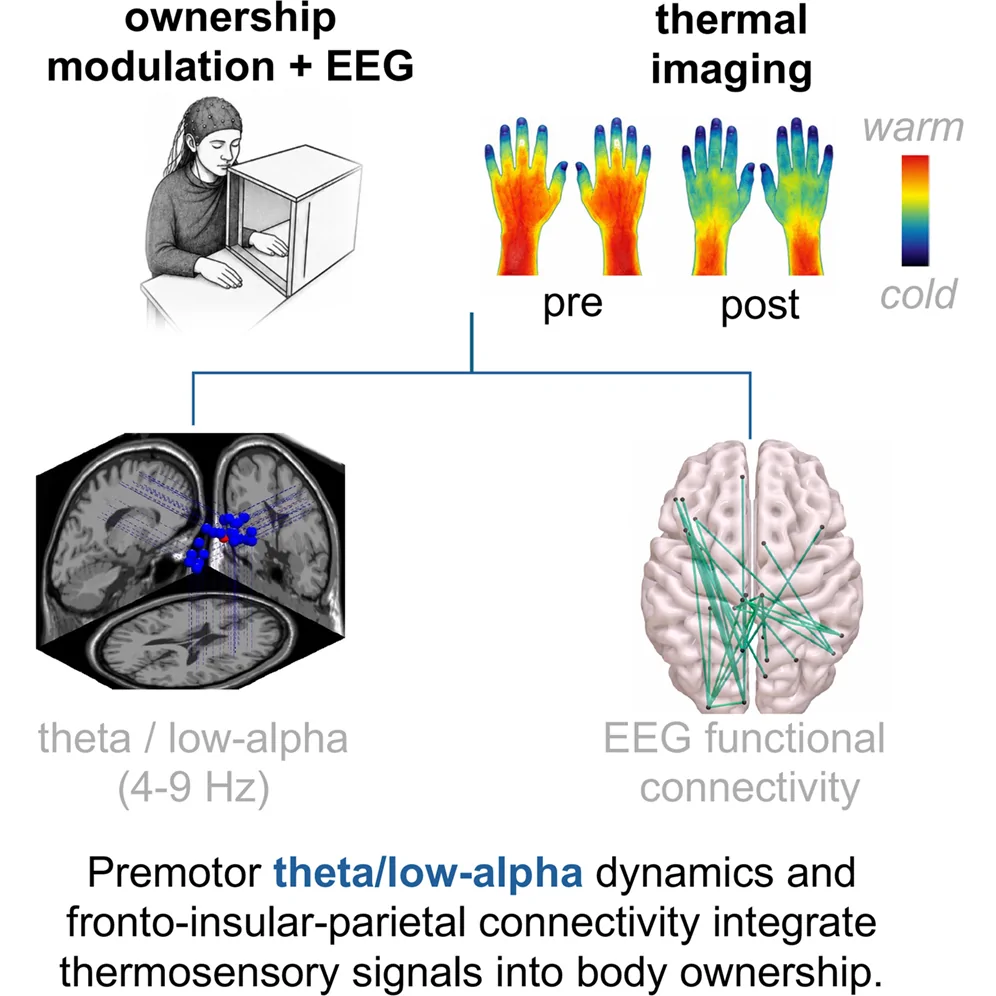

## Teaser

Skin temperature; Sensory neuroscience; Cognitive neuroscience; Machine perception

## Introduction

Skin temperature constitutes a fundamental physiological signal through which the brain continuously monitors the state of the body and its interaction with the environment.^1^^,^^2^ Rather than remaining static, cutaneous temperature dynamically fluctuates, providing continuous information that supports homeostasis and adaptive autonomic and behavioral responses essential for survival.^2^^,^^3^^,^^4^ Thermosensory signals arising from the skin ascend via the spinothalamic pathway and engage a distributed cortical network encompassing the insula, primary and secondary somatosensory cortices, parietal regions, and orbitofrontal cortex.^5^^,^^6^^,^^7^^,^^8^ Although skin temperature has traditionally been studied in the context of thermoregulation, accumulating evidence suggests that this signal is also associated with higher order representations of the self.^9^

A central component of bodily self-awareness is body ownership—the subjective experience that one’s body, or its constituent parts, belongs to oneself.^10^^,^^11^ Contemporary accounts conceptualize body ownership as an emergent cognitive representation of the dynamic integration of exteroceptive (e.g., vision), proprioceptive, motor, and interoceptive signals (e.g., heartbeat) within frontoparietal and insular networks.^12^^,^^13^^,^^14^ In healthy individuals, these integrative mechanisms have been predominantly investigated using multisensory illusion paradigms that transiently disrupt the coherence between sensory signals.^15^^,^^16^^,^^17^ By parametrically manipulating temporal and spatial congruency, such paradigms induce reliable changes in body ownership, providing experimental access to the dynamics associated with body ownership.^18^^,^^19^^,^^20^^,^^21^

A growing body of work shows that experimentally induced alterations in body ownership are accompanied by systematic changes in skin temperature (see for a review).^9^ Early studies employing the rubber hand illusion (RHI) reported decreases in skin temperature of the limb subjected to synchronous visuo-tactile stimulation,^22^^,^^23^ although subsequent investigations revealed substantial variability in the magnitude and reliability of these effects.^24^ Such discrepancies may reflect methodological differences across studies, including different experimental manipulations in the ownership paradigm, temperature acquisition techniques, and how temperature changes were quantified.^9^ For example, some studies have focused on temperature asymmetries between the two hands, an approach that may be less sensitive to bilateral temperature effects.^24^

More consistent findings have emerged from alternative paradigms. In the mirror box illusion (MBI), participants perform tapping movements with the index fingers of both hands while viewing the reflection of their right hand in a mirror positioned along the body midline, with the left hand hidden from view. During synchronous movements, participants may experience the reflected image of the right hand as if it were their own hidden left hand, thereby altering ownership of the unseen limb. Using this paradigm, our previous studies consistently reported bilateral reductions in skin temperature during the illusion.^25^^,^^26^^,^^27^ Similarly, full-body ownership illusions involving virtual avatars are associated with widespread decreases in body skin temperature,^28^ whereas reductions in disembodiment have been linked to increases in core gastrointestinal temperature.^29^

Clinical observations further underscore the functional relationship between skin temperature and body ownership. Abnormal temperature patterns have been documented in conditions characterized by disturbed bodily self-awareness, including complex regional pain syndrome,^30^ body integrity dysphoria,^31^ anorexia nervosa,^27^ and disturbed sensation of limb ownership (DSO) following right-hemisphere stroke.^32^ In particular, patients with DSO show reduced limb temperature, co-occurring with lesions involving the right insula and bilateral parietal disconnection, pointing to a large-scale bilateral network as a potential link between thermosensory processing and body ownership. Together, these findings indicate a robust association between skin temperature and body ownership, showing that this relationship extends beyond transient laboratory manipulations and may reflect a fundamental component of bodily self-awareness. However, they remain largely silent about the neural dynamics through which thermosensory signals are incorporated into the experience of owning a body.

Electrophysiological studies in healthy subjects have consistently reported that the illusion of ownership in multisensory paradigms is associated with reduced alpha/mu and beta power over central and parietal electrodes,^33^^,^^34^^,^^35^ increased gamma-band power, or phase synchrony in parietal-premotor regions during visuotactile congruence,^21^^,^^36^^,^^37^ and modulation of late theta activity in full-body and virtual-reality ownership illusions.^38^^,^^39^ Mirror-like paradigms have also been examined with electroencephalogram (EEG), revealing event-related desynchronization primarily in the alpha band over premotor and somatosensory cortices during synchronous visuomotor feedback.^40^^,^^41^^,^^42^^,^^43^ Critically, however, these studies have been confined to visual, tactile, and motor domains, offering no insight into the mechanisms linking thermosensory signals and the oscillatory dynamics underlying body ownership. Thus, although changes in skin temperature accompany ownership manipulations, the neural signature through which thermosensory signals shape bodily self-awareness remains an uncharted territory.

Here, we address this fundamental gap by combining behavioral measures, high-resolution thermal imaging, and electroencephalography within a unified multimodal framework. Using a well-established version of the MBI paradigm^16^^,^^25^^,^^26^^,^^27^—which quantifies body ownership through subjective vividness and proprioceptive drift and induces changes in skin temperature—we transiently altered ownership of a hidden hand in 36 healthy adults. In this paradigm, participants performed synchronous or asynchronous tapping movements with their left and right index fingers while viewing their right hand and its mirror reflection, with the left arm occluded from view. Importantly, the reflected image was spatially displaced from the actual location of the hidden left hand, creating a mismatch between visual and proprioceptive information. Relative to the asynchronous condition, synchronous stimulation typically induces the perception that the reflected right hand belongs to the left side (indexed by illusion vividness), a shift in perceived position of the hidden hand toward the reflected hand (proprioceptive drift), and a concomitant reduction in hand temperature measured via thermal imaging.

As suggested by previous literature, we expected that reductions in body ownership would be accompanied by decreases in hand temperature, potentially paired by selective modulations of oscillatory activity within a right-lateralized frontoparietal network.^33^^,^^40^^,^^42^ Furthermore, we anticipated that temperature-related fluctuations would be reflected in functional connectivity between temporo-parietal regions and the insula,^32^ thereby informing how thermosensory signals are dynamically integrated into neural systems that support bodily self-awareness.

## Results

### Behavioral

The MBI paradigm reliably altered body ownership, as reflected by increased perception of the right (reflected) hand as the left hand and by a spatial bias of the left index finger in the synchronous relative to the asynchronous condition (see Figure 1A).

**Figure 1:**
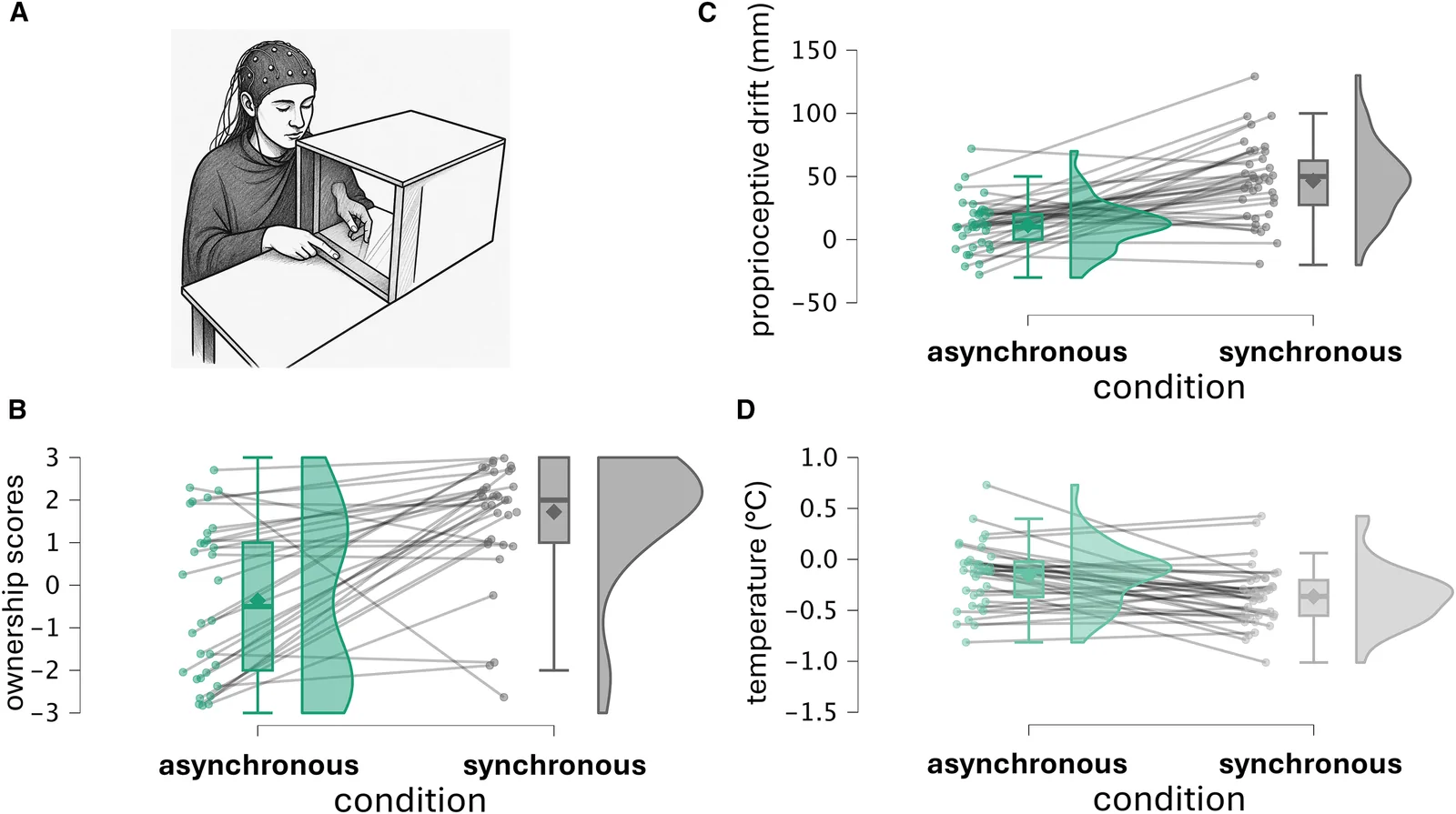
Experimental setup and results (A) Experimental setup: the participant sits in front of the mirror box, observing the reflection of their right hand. The participant’s left shoulder and arm are covered to prevent visual cues about the arm’s actual position inside the box. (B) Subjective ownership scores for the mirrored hand, with values from the synchronous condition in gray and those from the asynchronous condition in green. (C) Proprioceptive drift between the synchronous and asynchronous conditions is shown. (D) Average and observed delta temperature values (delta = post – pre-task thermal measurement) for both hands across the two conditions are shown. In each plot, individual observations are shown as dots, with lines connecting repeated measurements from the same participant. Boxplots display the interquartile range (IQR), with the horizontal line indicating the median and diamonds indicating the mean. Whiskers extend to the most extreme observations within 1.5 × IQR from the quartiles. Half-violin plots represent kernel density estimates of the data distribution.

#### Judgment of ownership

Synchronous stimulation was associated with a stronger participants’ perception of the reflected right hand as their left one (M = 1.722; SE = 0.240) compared to the asynchronous condition (M = –0.36; SE = 0.343; W = 351; *p* < 0.001; see Figure 1B).

#### Proprioceptive drift

Synchronous stimulation was associated with greater displacement of the perceived left-hand position toward the mirror compared to asynchronous stimulation (W = 527, *p* < 0.001; synchronous: M = 46.70 mm, SE = 5.16; asynchronous: M = 11.40 mm, SE = 3.36; Figure 1C).

### Thermal imaging

Synchronous stimulation was associated with a bilateral reduction in skin temperature relative to the asynchronous condition. Results showed a main effect of condition (F_(1,35)_ = 12.944, *p* < 0.001; η^2^_*p*_ = 0.270), indicating a decrease in the temperatures of both the left and right hands after the synchronous (M = −0.363°; SE = 0.049) compared to the asynchronous condition (M = −0.155C°; SE = 0.053). The main effect of side (F_(1,35)_ = 0.433; *p* = 0.515; η^2^_*p*_ = 0.012) and the interaction condition by side (F_(1,35)_ = 3.345; *p* = 0.216, η^2^_*p*_ = 0.087) were not significant (see Figure 1D). To examine the relationship between thermosensory and behavioral indices, we computed repeated-measures correlations (rmcorr) between temperature changes (post-pre-stimulation) and both ownership judgments and proprioceptive drift. A significant common within-individual association emerged between temperature changes and proprioceptive drift (r(34) = −0.381, 95% CI [−0.630, −0.059], *p* = 0.022), indicating that, across experimental conditions, larger reductions in skin temperature tended to covary with larger proprioceptive drift values within the same participant. No significant within-individual association was observed between temperature changes and ownership judgment ratings (*p* = 0.182).

### EEG: Independent components clustering

A right-lateralized frontoparietal cluster, with dipoles localized primarily in premotor/supplementary motor and parietal regions, showed differential oscillatory dynamics between synchronous and asynchronous conditions (*p* <0 05). Specifically, asynchronous stimulation was associated with greater event-related desynchronization in the theta/low-alpha band (4–9 Hz) around 300 ms after movement onset, whereas synchronous stimulation showed higher intertrial coherence in the low-theta band around 400 ms.

We next examined whether modulation of oscillatory activity within this cluster was associated with changes in skin temperature. The power spectra for synchronous and asynchronous conditions were extracted from the independent component (IC) included in the cluster (*n* = 23), using the frequency band showing significant event-related spectral perturbation (ERSP) modulation (i.e., 4–9 Hz). Then, a rmcorr was performed between the delta-temperature score (post-pre-stimulation) and theta/low-alpha power across the two experimental conditions. A significant common within-individual association emerged between theta/low-alpha power and temperature changes (r(22) = −0.488, 95% CI [−0.745, −0.105], *p* = 0.016; Figure 2), indicating that condition-related increases in theta/low-alpha power tended to covary with larger reductions in skin temperature within the same participant. Notably, theta/low-alpha power was not significantly associated with ownership judgment ratings or proprioceptive drift (ps > 0.05).

**Figure 2:**
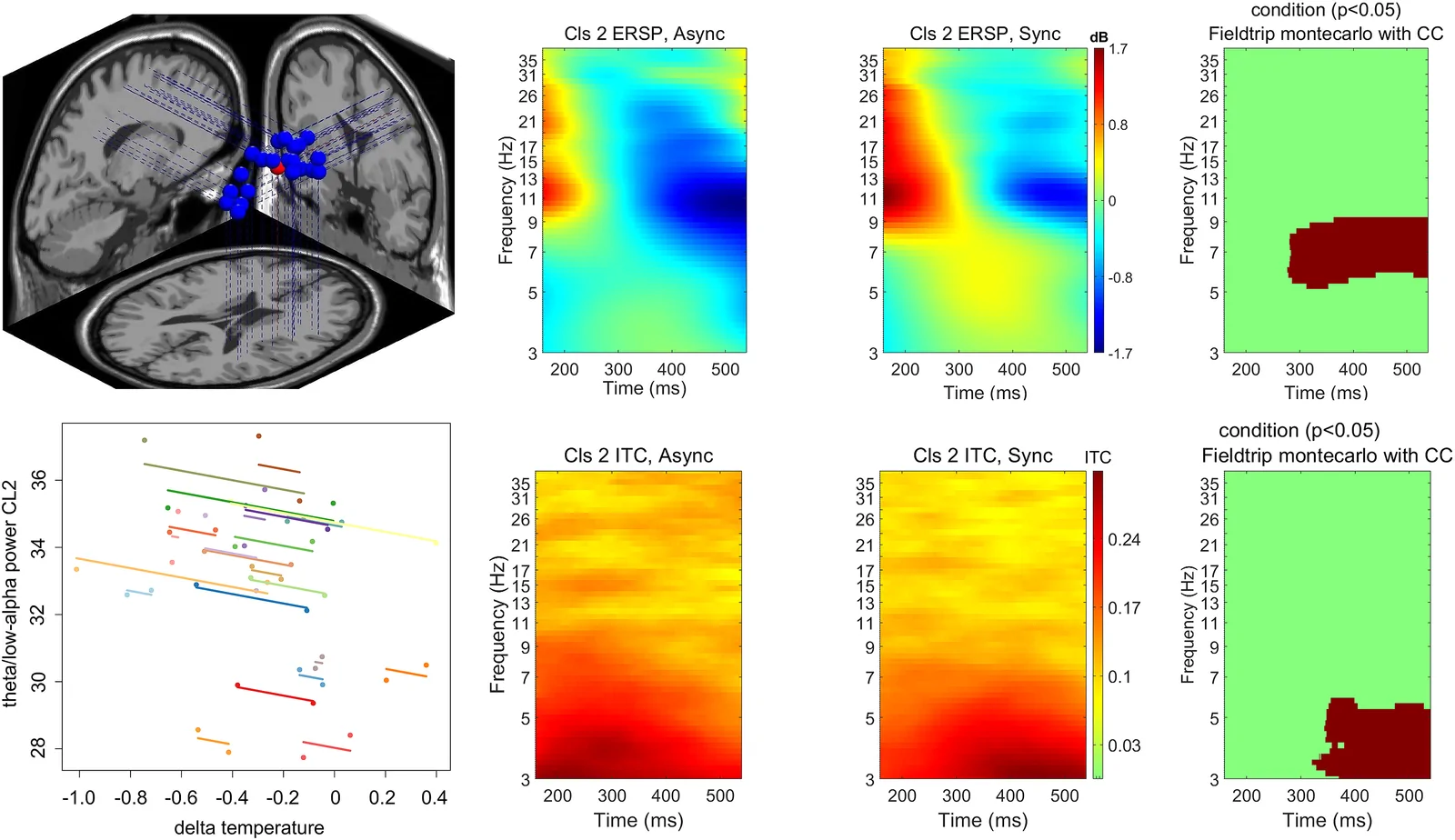
EEG results Dipoles of the ICs cluster 2 (Cl 2; left, top) showing significant differences in ERSP (right, top) and ITC (left, bottom) between asynchronous and synchronous conditions of the MBI. Left, bottom: correlation between delta temperature values and the theta-low alpha power values of the cluster across the two conditions for each participant.

### EEG functional connectivity

Contemporary models of thermal physiology highlight that variations in skin temperature involve multiple brain regions, such as the parietal, sensorimotor, and insular cortices.^7^^,^^8^^,^^44^ Therefore, to assess whether the local theta/low-alpha effects identified by the IC analysis were accompanied by potentially larger scale network interactions, source-level functional connectivity was computed in the same frequency range showing significant ERSP modulation (i.e., theta/low-alpha band, 4–9 Hz) using the debiased weighted phase lag index (WPLI)^45^ as a connectivity metric. A clustering analysis of connections significantly greater in the synchronous as compared to the asynchronous conditions identified six distinct connectivity patterns induced by the MBI procedure (Figure S2). Notably, a distributed frontoparietal-insular network, including bilateral sensorimotor cortices, superior parietal regions, the right supramarginal gyrus, and the left anterior insula/operculum, was associated with temperature changes. Within the same theta/low-alpha frequency range identified by the IC analysis, stronger connectivity within this network was associated with greater reductions in skin temperature (*r*_(34)_ = −0.35; *p* = 0.035; Figure 3). No connectivity patterns significantly correlated with the same measures for the opposite contrast (i.e., connectivity in the asynchronous condition was greater than in the synchronous condition, see supplemental information).

**Figure 3:**
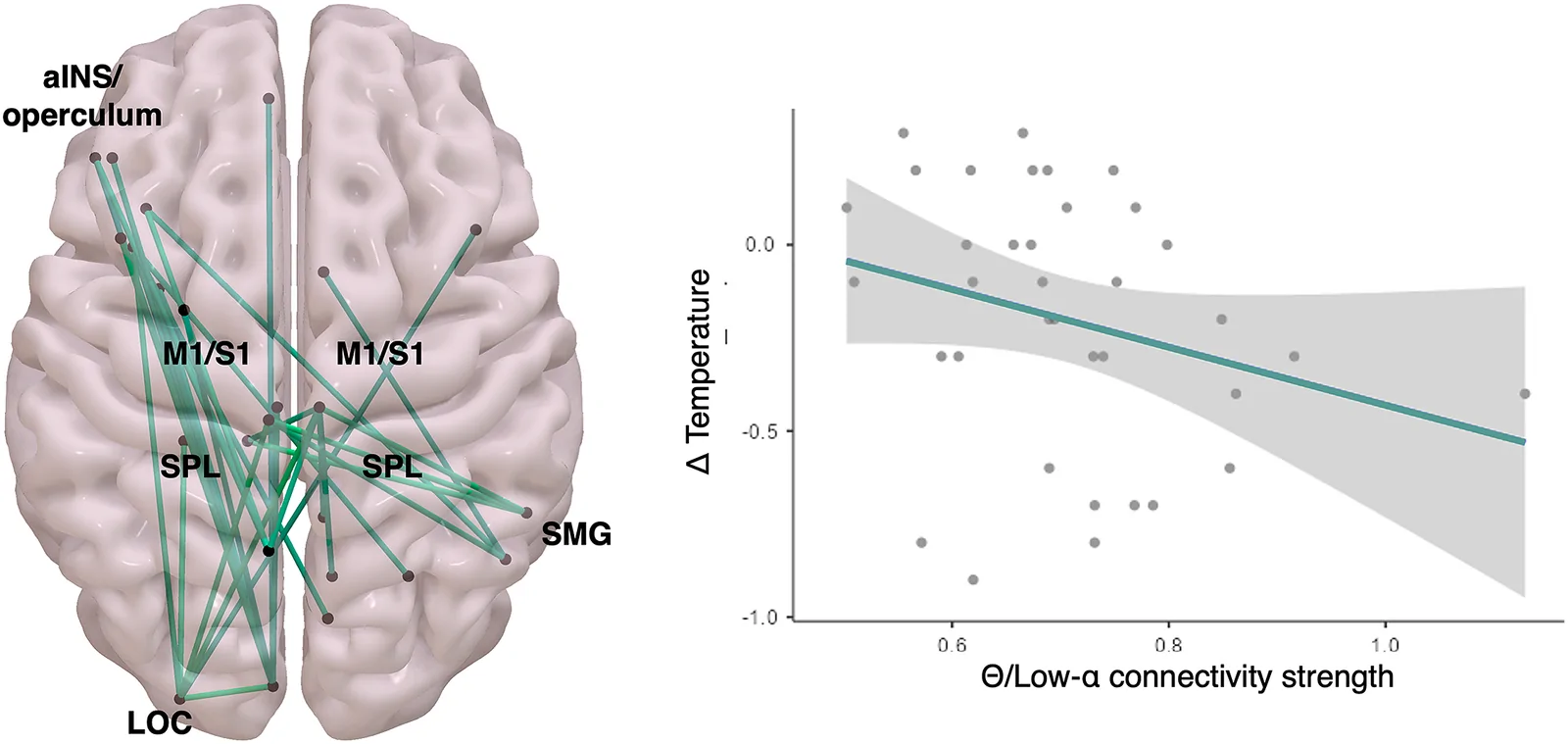
Connectivity module in the theta-low alpha bands (4–9 Hz) Bilateral temporo-parietal-insular connectivity module (left) and correlation of its connectivity strength with temperature decrease (right). Stronger connections within the module during the mirror-box procedure correlate with a greater subsequent drop in hand temperature.

## Discussion

Hand temperature has been suggested as a potential physiological factor contributing to the construction of the sense of ownership.^22^^,^^23^^,^^25^^,^^26^^,^^27^ However, the neural dynamics linking thermosensory signals and body ownership remain unknown. Here, by combining electroencephalography with thermal imaging during a MBI, we identify oscillatory dynamics associated with thermosensory changes during altered body ownership. A large-scale network, including frontal, parietal, occipital, and insular areas, may support the coherent integration of the physiological body into the mental self-representation.^46^

Our findings show that transient alterations of hand ownership are associated with right-lateralized frontoparietal dynamics, whose low-frequency oscillatory activity tracks changes in hand temperature. Both synchronous and asynchronous mirror-box stimulation engaged a right-lateralized premotor-parietal network characterized by theta/low-alpha (4–9 Hz) activity. While asynchronous stimulation produced stronger theta/low-alpha desynchronization, synchronous stimulation was associated with greater inter-trial phase consistency within the same network. This may suggest that the visuo-motor mismatch in the asynchronous condition requires greater engagement of sensorimotor areas to process the incongruence. The synchronous condition may instead reduce the sensorimotor computational load by providing an integrated and coherent multisensory signal, resulting in less desynchronization compared to active mismatch.^37^^,^^47^

Notably, these theta/low-alpha modulations covaried with reductions in skin temperature, whereas no significant associations emerged with subjective vividness or proprioceptive drift. This pattern is consistent with the view that body ownership emerges from distributed neural systems that support partially distinct aspects of bodily self-representation.^12^^,^^13^ In this regard, classic lesion studies in non-human primates offer a plausible mechanistic account. Kessler et al.^48^ reported that lesions involving premotor and posterior parietal cortices produced persistent reductions in the temperature of the contralateral limb, despite the absence of primary autonomic damage. These findings led the authors to propose that cortical regions traditionally associated with sensorimotor integration also contribute to the regulation of peripheral vasomotor responses. The present findings extend this observation by showing that activity within frontoparietal regions covaries with temperature changes during an ownership manipulation, suggesting a possible link between body ownership and thermosensory signals in these cerebral regions.

The present findings also extend prior evidence implicating premotor and parietal circuits in multisensory integration and recalibration of body ownership indices.^42^^,^^49^ In a similar task investigating the EEG correlates of the mirror phenomenon, participants were asked to perform unilateral movements in front of a mirror to induce the illusion of bilateral synchronous limb movement. The illusion was associated with event-related desynchronization of alpha (8–12 Hz) rhythms at central and parietal scalp electrodes ipsilateral to unilateral right finger movements.^41^ The illusory condition was characterized by greater alpha event-related desynchronization in right premotor, primary somatomotor, and posterior inferior parietal areas.^42^ Although the direction of the oscillatory effect differs from the present findings, the paradigms are not directly comparable. Previous studies contrasted mirror visual feedback with non-illusion control conditions, whereas the present study compared synchronous and asynchronous mirror feedback. Despite these differences, both sets of findings implicate premotor and parietal regions in multisensory processes related to body ownership.

In this study, by jointly quantifying cognitive and physiological indices of ownership, we extend previous work by defining the functional relevance of these oscillatory dynamics. Interestingly, the link between lower skin temperature and these oscillatory effects suggests that temperature-related changes are associated with both local oscillatory dynamics and large-scale network interactions operating within the same theta/low-alpha frequency range. Moreover, although the oscillatory effect was predominantly right-lateralized, bilateral temperature reductions are not inconsistent with this finding. Previous studies in both healthy individuals and those with right-brain damage have shown that unilateral manipulations or disturbances of body ownership are accompanied by bilateral temperature changes.^25^^,^^26^^,^^27^^,^^31^^,^^32^^,^^50^^,^^51^ One possible explanation lies in the right hemisphere’s specialization for body ownership, which maintains a representation of bilateral somatosensory signals (e.g., touch^52^), and, in the case of temperature, is potentially sustained via interhemispheric connections, as demonstrated by the only available lesional study.^32^ Indeed, contemporary thermal physiology models emphasize that changes in skin temperature may involve a bilateral network, including the parietal, sensorimotor, and insular cortices.^7^^,^^44^^,^^53^ In line with this framework, our EEG connectivity analyses revealed that reductions in skin temperature were associated with increased low-frequency coupling across a distributed frontoparietal-insular network, encompassing bilateral sensorimotor cortices, superior parietal regions, the right supramarginal gyrus, and the left anterior insula/operculum. Notably, this network partially overlaps with circuitry implicated in the relationship between thermosensory signals and body ownership in clinical populations, as found in a previous study from our group on stroke patients with disturbed ownership.^32^ A connectome-based lesion-symptom mapping analysis revealed that the observed bilateral hand temperature decrease in patients with disownership was associated with the disconnection of three hub-like structures: the right primary somatosensory cortex, the secondary somatosensory cortex, and the inferior parietal gyrus, which were disconnected mainly from their contralateral homologous areas. The correspondence between network-level dynamics observed here in healthy participants and patterns of disruption reported in clinical conditions suggests that thermosensory signals constitute an integral component of the neural architecture supporting bodily self-awareness, rather than an epiphenomenal consequence of ownership alterations.

These findings inform theoretical accounts linking thermosensory processing and body ownership.^9^ Predictive frameworks propose that ownership illusions engage sensory reweighting processes that accompany multisensory conflict. In this context, visual information may be prioritized over competing somatosensory signals during body ownership updates. Previous work has shown that other somatosensory signals, such as touch, can be downweighted during ownership illusions.^54^ Although the physiological mechanisms underlying thermal and tactile signals are likely distinct, thermosensory signals may also be affected during multisensory conflict. Importantly, the present findings identify oscillatory and connectivity patterns that provide complementary perspectives on this effect, highlighting both local frontoparietal oscillatory dynamics and large-scale frontoparietal-insular interactions within the theta/low-alpha range. Future studies will be necessary to determine the exact mechanisms underlying these effects.

An additional open question concerns the temporal dynamics of these phenomena. While subjective ownership may emerge rapidly during multisensory stimulation, temperature changes are likely to evolve more gradually. Future research combining continuous temperature monitoring with online measures of ownership could help clarify the relative time course and causal relationship between these processes.

Taken together, our findings open promising avenues for future research, such as exploring thermosensory impairments and their potential effects on body ownership, developing advanced prosthetic systems that incorporate thermosensory feedback,^55^ and examining how healthy and pathological aging affect the thermosensory system and self-awareness. Moreover, evidence suggests that thermosensory manipulations may have therapeutic potential. Previous studies have shown that increasing limb temperature can strengthen body ownership in healthy individuals^56^ and correlate with the recovery of ownership disturbances in a patient with somatoparaphrenia.^51^ Understanding the neural mechanisms linking thermosensory signals and body ownership may therefore inform the development of novel rehabilitation strategies targeting disorders of bodily self-awareness.

### Limitations of the study

Although the present study provides novel evidence linking thermosensory changes to oscillatory dynamics during body ownership modulation, several limitations should be acknowledged. First, the sample consisted exclusively of healthy young adults, which may limit the generalizability of the findings to other populations. Second, although EEG source reconstruction and connectivity analyses suggested the involvement of a frontoparietal-insular network, the spatial resolution of EEG does not allow definitive conclusions regarding the precise anatomical generators of the observed effects. Third, hand temperature was measured before and after each experimental condition rather than continuously throughout the task, preventing a fine-grained characterization of the temporal relationship between thermosensory fluctuations and ownership-related neural dynamics. Finally, the correlational nature of the associations between temperature changes and oscillatory activity precludes causal inferences about whether thermosensory signals directly contribute to body ownership updates. Future studies combining continuous temperature monitoring, fMRI, and experimental manipulation of thermosensory signals will be necessary to address these issues.

## Resource availability

### Lead contact

Requests for further information and resources should be directed to and will be fulfilled by the lead contact, Gerardo Salvato (gerardo.salvato@unipv.it).

### Materials availability

No materials were generated in the study.

### Data and code availability

• All data reported in this paper will be shared by the lead contact upon request.
• This paper does not report original code.
• Any additional information required to reanalyze the data reported in this paper is available from the lead contact upon request.

## Author contributions

Conceptualization, G.M., F.C., and G.S.; methodology, G.M., F.C., A.P., and G.S.; investigation, F.C., I.G., and C.M.; formal analysis, G.M., F.C., I.G., C.M., E.A., and A.P.; writing – original draft, G.M., F.C., and G.S.; writing – review & editing, all authors; supervision, G.B. and G.S.

## Declaration of interests

The authors declare no competing interests.

## STAR★Methods

### Key resources table

**Table:** 

REAGENT or RESOURCE	SOURCE	IDENTIFIER
**Software and algorithms**		

JAMOVI	The jamovi project	https://www.jamovi.org/ Version2.6.2.0
R	R core team	https://cran.r-project.org/ Version 4.3.1
G∗Power 3.1	Faul et al.^57^	https://www.psychologie.hhu.de/arbeitsgruppen/allgemeine-psychologie-und-arbeitspsychologie/gpower
MATLAB	MathWorks	R2018b
EEGLAB	Delorme & Makeig^58^	https://eeglab.org
Fieldtrip Toolbox	Oostenveld et al.^59^	https://www.fieldtriptoolbox.org
E-prime 3	Psychology Software Tools Inc.	https://pstnet.com/products/e-prime/ Version3.0.3.80
FLIR Research Studio	Teledyne FLIR LLC., Wilsonville, OR, USA	https://www.flir.com/it-it/products/flir-research-studio/
Brain Connectivity toolbox	Rubinov & Sporns^60^	https://sites.google.com/site/bctnet/home?authuser=0
Surf Ice	Rorden^61^	https://www.nitrc.org/projects/surfice/

**Other**		

FLIR E−76 digital infrared camera	FLIR Teledyne System, TM	https://www.flir.com
EEG cap and amplifier	g.tec medical engineering GmbH, Austria	https://www.gtec.at

### Experimental model and study participant details

#### Human participants

We hypothesized a between-conditions difference in hand skin temperature, with α = .05 and power (1−β) = .95, using a two-dependent-means two-tailed t-test. The power analysis was based on pilot data reported in our previous study,^26^ which employed the same mirror box illusion paradigm and the same hand-temperature outcome measure used in the present experiment. The observed temperature difference between the synchronous and asynchronous conditions corresponded to a Cohen's d_z_ of 0.62. Based on this effect size, an *a priori* power analysis performed with G∗Power 3.1^57^ indicated a required sample size of 36 participants.

Thirty-six healthy volunteers [26 females (age range: 18–47 years, *M* = 24.58, *SD* = 5.23); education: range 13–22 years, *M* = 15.26, *SD* = 2.62] participated in the study. All participants were native Italian speakers, had normal or corrected-to-normal vision, and had no previous history of mental or neurological illness. None of the participants reported peripheral nervous system pathology (e.g., Raynaud’s syndrome). We administered the Edinburgh Handedness Inventory (EHI)^62^ to ensure all the participants were right-handed. They received course credit for their involvement. We did not evaluate potential effects of sex and gender in the present study; we acknowledge this limitation, which may restrict the generalizability of the results. Before the experiment, all participants provided written informed consent. The Institutional Ethics Committee of Istituti Clinici Scientifici Maugeri IRCCS, Pavia, approved the experimental procedures (Protocol number 2197), which comply with the Declaration of Helsinki.

### Method details

#### Mirror box illusion paradigm

##### Apparatus

We used the same experimental paradigm as in previous studies.^16^^,^^25^^,^^26^^,^^27^ The mirror box was built using a flat wooden board (91.4 cm long × 40.6 cm wide) with two acrylic mirrors (40.6 cm wide × 30.5 cm high) placed in the middle. The two mirrors were 15.24 cm (6′) apart. Participants had their right and left hands positioned against the mirrors. To limit visual cues about the left arm's actual position, a cloak was placed over the subject's left shoulder. On the upper part of the box, perpendicular to the mirrors, a modified meter paper strip (20.24 cm long, starting 5 cm from the left, at the actual position of the covered mirror, and ending 15 cm to the right) was visible to the participants.

##### Task

Participants were instructed to tap their right and left index fingers against the mirrors while focusing on the reflection of their right hand, with the left hand occluded.

In the synchronous condition, participants tapped both index fingers simultaneously. In the asynchronous condition, participants tapped at the same tempo, but the two index fingers moved simultaneously in opposite directions: while one finger moved toward the mirror, the other moved away from it. Thus, movement frequency and the total number of movements were matched across conditions, while only visuomotor congruence differed. Each condition consisted of one block with the same number of trials (*N*=100), with one tapping movement per trial, triggered by a standardized sound signal delivered through a computer speaker. E-prime software (Version 3.0.3.80) was used to deliver a triggering sound, with the intertrial interval jittered between 1500 and 2000 milliseconds, and to code the onset of trials in the EEG continuous recording. Each condition lasted approximately 3 minutes, with a 15-minute interval between conditions to promote a more stable participant acclimatization before the subsequent condition. In addition, the order of the conditions was counterbalanced to minimize potential effects on skin temperature related to time spent in the experimental room.

##### Judgement of ownership

We investigated participants' explicit judgement of ownership (i.e., the sense of ownership towards the reflected hand) using a statement from the Embodiment Questionnaire used in previous applications of the MBI paradigm.^25^^,^^26^ Participants indicated their level of agreement with the statement *“It felt as though the hand in the mirror was my left hand”* on a seven-point Likert scale, from +3 (completely agree) to −3 (completely disagree).

##### Proprioceptive drift

Participants were asked to verbally localize the position of the tip of their left index finger using the ruler attached to the top of the box (i.e., proprioceptive drift) before and after each experimental condition. Verbal reports were used to avoid introducing additional proprioceptive feedback associated with pointing movements. The ruler displayed scrambled numbers and remained covered throughout the experimental condition, reducing the possibility that participants relied on previously reported locations rather than their current perceived finger position.

#### Thermal imaging

Before and after each experimental condition, we measured participants' hand temperatures using thermal imaging. We took three consecutive thermal images immediately before and after each condition of the MBI task using a FLIR E-76 digital infrared camera (FLIR Teledyne System, TM). The camera was placed on a tripod behind the mirror box, at a fixed distance of 70 cm from the mirror box surface. The thermographic readings were obtained using FLIR Thermal Studio Pro software (FLIR Teledyne System, TM), which allows drawing polygonal Regions of Interest (ROI) on the hand thermographic image, adaptable to the individual subject. To maximize measurement reliability, we drew the ROI set for each participant and exported it to subsequent images. Infrared thermal images for each participant were divided into two separate ROIs corresponding to the dorsal surfaces of the right and left hands. The software calculated the mean pixel value within each drawn polygon, yielding the average surface temperature for each ROI.

To minimize possible confounding variables for the measurements of skin temperature, before participating in the experiment, subjects were instructed to: i) avoid drinking alcohol, theine, or caffeine for at least 24 h; ii) avoid using cosmetic products on both hands for at least 24 h; and iii) keep their eyes closed throughout the temperature measurements of the body parts. Furthermore, the measured body parts were not in contact with any external objects (e.g., a phone), and the experimenter did not contact the subjects' body parts. In addition, during the experiment, thermal exchanges and electromagnetic interference with the infrared medical device were avoided by switching off the electronic devices (e.g., cell phones) in the room. Furthermore, to minimize fluctuations in body temperature, all experimental sessions were conducted in the same shielded, air-conditioned EEG cabin, ensuring that ambient temperature and relative humidity remained as stable as possible across participants and experimental conditions. In addition, participants were acclimatized for at least 15 minutes before the start of the experiment, which corresponded to the time required for EEG headset preparation and mounting.

#### EEG recording and data preprocessing

EEG was continuously recorded with 64 passive electrodes (g.tec medical engineering GmbH, Austria) at 1200 Hz sampling rate, maintaining between electrodes impedance below 5 kΩ. Electrode placement was based on the 10-20 international system, using FPZ as the ground and CZ as the online reference electrode. Following data collection, raw EEG data were imported into MATLAB and preprocessed using EEGLAB (v. 2018b).^58^ Data were band-pass filtered between 1 and 40 Hz, resampled to 600 Hz. Removed channels were reconstructed using spherical interpolation based on weighted averages of neighboring channels. A small number of channels required interpolation (mean = 0.14 channels per participant, range = 0–2). Signal was re-referenced to the common average reference, and the continuous signal was segmented into epochs from -400 ms to +1100 ms relative to the auditory cue. The epoch duration was selected to avoid overlap with subsequent trials, even for the shortest inter-stimulus interval (1500 ms), and to provide an adequate prestimulus baseline period. For ERSP and ITC analyses, baseline correction was applied using the prestimulus interval (-400 to 0 ms), and a common baseline across conditions was used for statistical comparisons. Trials with excessive noise (i.e., ocular or muscular artifacts) were manually rejected based on visual inspection. Out of 100 trials per condition, an average of 99.39 (SD = 0.84) and 99.42 (SD = 0.91) trials were retained for analysis in the synchronous and asynchronous conditions, respectively. Then, signal decomposition was performed using Independent Component Analysis (ICA) with the runica algorithm^63^^,^^64^ and the ICLabel function in EEGLAB to discard non-brain sources from the EEG signal and classify components (e.g., eyeblinks, channel noise). Noise-related ICs were further refined by visual inspection. Subsequently, the DIPFIT function was used to estimate a single-equivalent-current dipole model for each IC, fitting dipole sources within a co-registered boundary-element head model in MNI space and localizing ICs using eLoreta.^65^^,^^66^^,^^67^

### Quantification and statistical analysis

#### Behavioral and thermographic imaging analyses

We first verified that the MBI paradigm successfully modulated ownership by comparing vividness ratings (judgment of ownership) and proprioceptive drift (in mm) between the Synchronous and Asynchronous conditions using paired Wilcoxon signed-rank tests. We then evaluated potential changes in skin temperature across experimental conditions and laterality. We used a repeated measure ANOVA with Condition (Synchronous, Asynchronous) and Side (Left, Right) as factors. The delta values between the ROI mean temperature pre- and post-task were modelled as the dependent variable. Data analyses were conducted using JAMOVI Version 2.6.2.0. To assess the relationship between thermosensory and behavioral indices of body ownership, a repeated-measures correlation (*rmcorr*) was performed between temperature change and judgment of ownership and proprioceptive drift across experimental conditions. This approach estimates the common within-individual association between the two variables while accounting for the non-independence of repeated observations and controlling for stable between-subject differences.^68^ For these analyses, we used *R* Version 4.3.1.

#### EEG analyses: Independent component clustering and spectral analyses

Principal component analysis (PCA) implemented in EEGLAB was applied for pre-clustering ICs. Only ICs with less than 15% of dipole models’ residual variance were included in clusters for group analyses. The pre-clustering matrix was built based on spatial features of dipoles (locations, orientations and scalp maps). Subsequently, k-means clustering was performed. Following the default EEGLAB settings, the number of clusters was automatically determined such that, on average, approximately one IC per participant was assigned to each cluster, resulting in K = 15 clusters for the present dataset. The clusters resulting from this automatic procedure were then manually refined by visual inspection, keeping only one IC for each participant within each cluster, selecting ICs with the lowest residual variance and most consistent dipole location and scalp topography.^69^^,^^70^

The cluster analysis yielded 15 independent component (IC) clusters. Clusters were selected for statistical analysis based on consistency of dipole localization within task-related brain regions and on cross-subject representativeness, operationalized as the inclusion of ICs from at least 60% of participants.^71^^,^^72^ Three clusters met these criteria, containing dipoles in task-related brain regions and including ICs from at least 22 participants (60% of the total sample; see Supplementary Material for cluster details), and were therefore retained for statistical analysis of EEG time–frequency dynamics to assess the effect of MBI on Event-Related Spectral Perturbation (ERSP) and Inter-Trial Coherence (ITC).

Time-frequency analyses were carried out on ICA components using EEGLAB’s functions. Spectral decomposition was computed on a single-trial basis and then averaged across trials, using Morlet wavelets with 3 cycles at the lowest frequency, increasing with frequency scaling factor 0.8, across 100 logarithmically spaced frequencies, and 200 output time points. ERSP was obtained by averaging single-trial power estimates across trials, and ITC was computed as the consistency of phase across trials from the complex time–frequency estimates.

Statistical analyses were performed using Fieldtrip-based permutation testing with cluster-level correction as implemented in EEGLAB, applying a common prestimulus baseline (−400 to 0 ms) across conditions for ERSP analyses. Cluster-level correction was applied using Monte Carlo permutation testing (10000 permutations), with a cluster-forming threshold of *p* < 0.05 and cluster mass as the cluster statistic.

Lastly, to assess the relationship between thermosensory changes and neural activity, repeated-measures correlations (rmcorr) were performed between temperature change and the oscillatory power of the IC clusters identified in the ERSP analyses. This approach estimates the common within-individual association between physiological and electrophysiological measures across experimental conditions while accounting for repeated observations within participants and controlling for stable between-subject differences.^68^

#### Connectivity analyses: Source reconstruction

Source reconstruction was performed with Fieldtrip.^59^ The forward model was created starting from a standard Boundary Element Method (BEM) volume conduction model available in Fieldtrip. In detail, the head model was created by assigning standard conductivity values for the scalp, skull, and brain compartments.^73^^,^^74^ Lead-field matrices were created by aligning the forward model with standard 10-10 system electrode positions. To estimate the data covariance required for spatial filtering, sensor-level covariance matrices were computed. A Linearly Constrained Minimum Variance (LCMV) beamformer was used as the inverse method. A regularization parameter of 5% was applied. Dipole orientations were constrained to be fixed.

Following source reconstruction, source-level time series were obtained by projecting the sensor-level data through the LCMV spatial filters. For each parcel in the source model, the corresponding spatial filter was applied to the preprocessed sensor data, yielding parcel-wise source-space signals. Source signals were segmented into 100 regions of interest (ROIs) according to the Schaefer atlas.^75^ Spectral decomposition of the parcel-wise source data was performed using a multitaper fast Fourier transform implemented in Fieldtrip. Spectral estimates were obtained for the frequencies of interest ranging between 5 and 9 Hz. Then, functional connectivity between all pairs of parcels was computed in the frequency domain using the debiased Weighted Phase Lag Index (wPLI-debiased).^45^ This metric quantifies phase synchronization while reducing bias associated with sample size and minimizing the contribution of zero-lag interactions. wPLI values were averaged across frequency bins to derive subject-level connectivity matrices for each experimental condition (i.e., synchronous, asynchronous).

At the group level, connectivity matrices derived from the two experimental conditions were exploratively statistically contrasted at the edge level. Debiased wPLI matrices from the synchronous and asynchronous mirror box conditions were compared using a one-tailed pairwise permutation-corrected t-test with an alpha level of .05 (10000 permutations^76^), testing first the hypothesis that connectivity was stronger in the synchronous condition and then the opposite hypothesis (see^21^^,^^77^^,^^78^^,^^79^ for similar procedures). Only connections surviving this statistical thresholding were retained for subsequent network analysis. Results are visualised using Surf Ice.^61^ To characterize large-scale functional organization, modularity was quantified using the Brain Connectivity Toolbox,^60^ employing the modularity_und function for undirected networks. This procedure identified community structure based on patterns of significant connections that differed between conditions. For each detected module, within-module connectivity strength was computed by summing all WPLI values corresponding to edges whose endpoints belonged to that module. These module-level strength values were then subjected to correlation analyses. Namely, for each module, connectivity strength was correlated with thermal (i.e., delta temperature asynchronous-synchronous) and behavioral measures of ownership (i.e., delta proprioceptive drift asynchronous-synchronous), and embodiment (i.e., delta ownership asynchronous-synchronous) to assess the relationship between network-level synchronization during the mirror box illusion induction and its effects at interoceptive and proprioceptive levels. As the thresholding procedure produces different patterns for each contrast (i.e., synchronous>asynchronous and vice versa), a similarity in network strength between these conditions would not be balanced, preventing the rmcorr approach. For this reason, classic Pearson correlations were performed separately for synchronous and asynchronous network strength values.
