# Supplementary material for: Oscillatory dynamics within fronto-insular-parietal networks integrate thermosensory signals into body ownership
Source: iScience. 2026 Sep 26;29(10):117679. doi: 10.1016/j.isci.2026.117679 (PMC13638793; doi:10.1016/j.isci.2026.117679)
Supplement: Supplementary file 1 [file mmc1.pdf]

iScience, Volume *29*

## Supplemental information

# Oscillatory dynamics within fronto-insular-parietal networks integrate thermosensory signals into body ownership

**Giulia Mattavelli, Francesco Crottini, Irene Gorrino, Eleonora Arrigoni, Carlotta Maiocchi, Alberto Pisoni, Gabriella Bottini, and Gerardo Salvato**

**Table S1.** Clusters resulting from ICA. Bold font highlights clusters retained for statistical analysis.

| Cluster number | Cluster location | Number of ICs | x y z coordinates | RV |
|---|---|---|---|---|
| **2** | **right premotor/supplementary motor and parietal areas** | **23** | **33.1(10.1) -5.5(21.3) 45.4(8.1)** | **6.03%** |
| **6** | **Secondary visual/visuomotor /dorsal posterior cingulate cortex** | **28** | **1.6(7.9) -65.3(16.1) 29.7(14.2)** | **4.7%** |
| **15** | **Anterior cingulate cortex/basal ganglia/bilateral premotor premotor cortex** | **27** | **-1.5(16.9) 3.2(10.6) 27.4(15.2)** | **4.81%** |
| 1 | Outside defined BAs | 19 | -2.9(13.4) -1.5(11.4) 22.3(15.1) | 8.72% |
| 3 | Left Secondary Visual cortex | 20 | -23.2(14.3) -89(10.4) -16.6(11.8) | 4.81% |
| 4 | Outside defined BAs | 16 | 5.3(16.1) -22(26.5) -4(17) | 8.56% |
| 5 | Left Primary Motor Cortex | 19 | -35.3(9.6) -9.6(17.6) 40.4(11.7) | 4.65% |
| 7 | Outside defined BAs | 19 | 34.7(10.8) -69.4(11.4) 5.5(11.6) | 5.03% |
| 8 | Outside defined BAs | 19 | -26.6(13.7) -50.9(13.3) 12.3(15.6) | 7.1% |
| 9 | Right Ventral Posterior Cingulate cortex | 20 | 5.85(16.9) -18.1(19.1) 30(14.6) | 8.95% |
| 10 | Outside defined BAs | 17 | 8.6(22.4) 25.9(17.4) 9.9(13) | 7.4% |
| 11 | Right Orbitofrontal Cortex | 3 | 1.7(3.8) 21.3(4) -23.3(1.5) | 9.82% |
| 12 | Left Orbitofrontal Cortex | 13 | -1.9(16.5) 23.7(15.4) -18.5(7) | 8.97% |
| 13 | Left Middle Temporal Gyrus | 19 | -49.2(11.2) -18.4(26.5) -15(16) | 6.52% |
| 14 | Right Parahippocampal Gyrus | 17 | 19.8(14) -26.5(14) -4.1(21.2) | 8.46% |

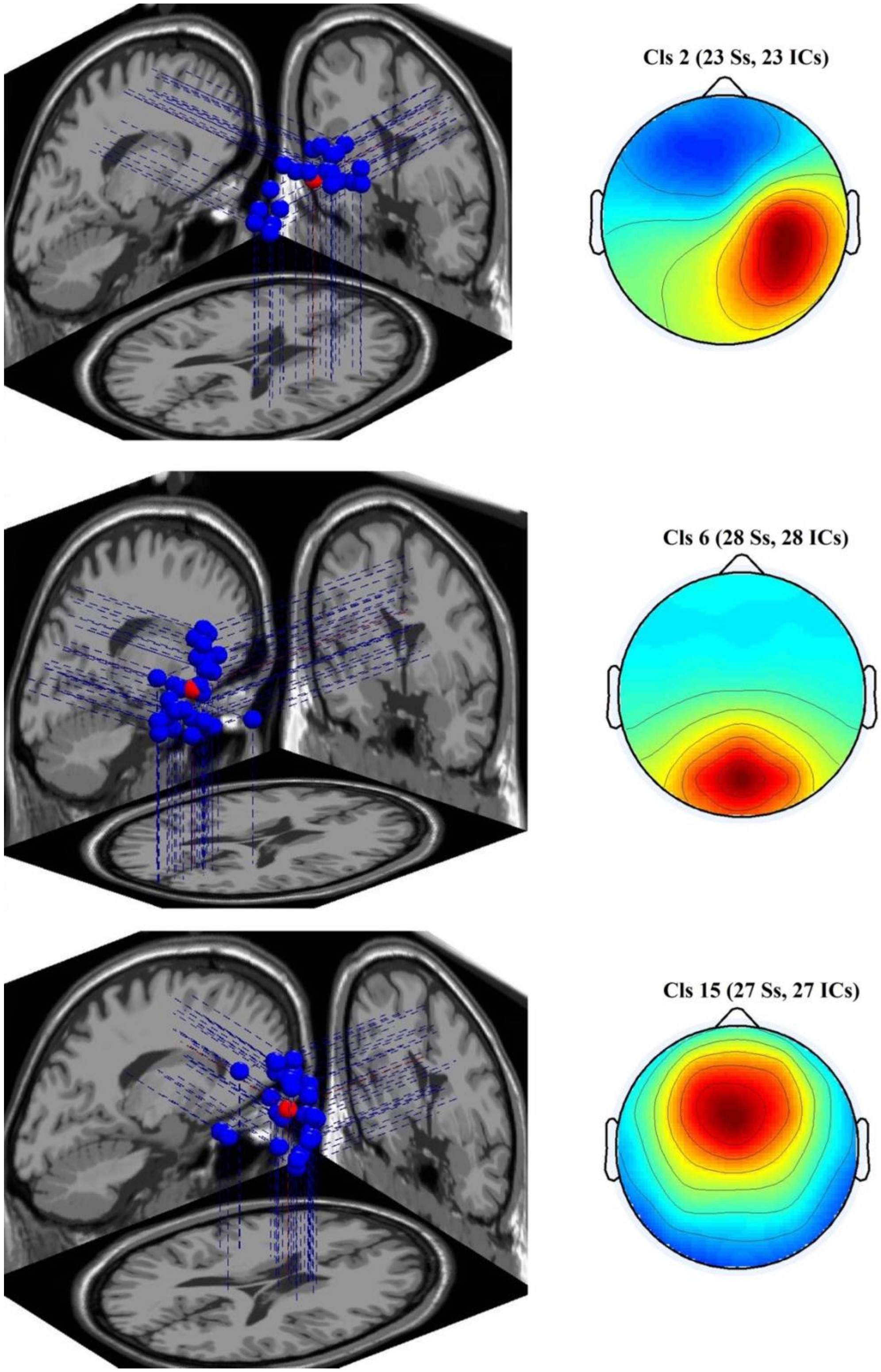


**Fig S1.** Cluster dipoles and topography of the three clusters retained for statistical analysis.

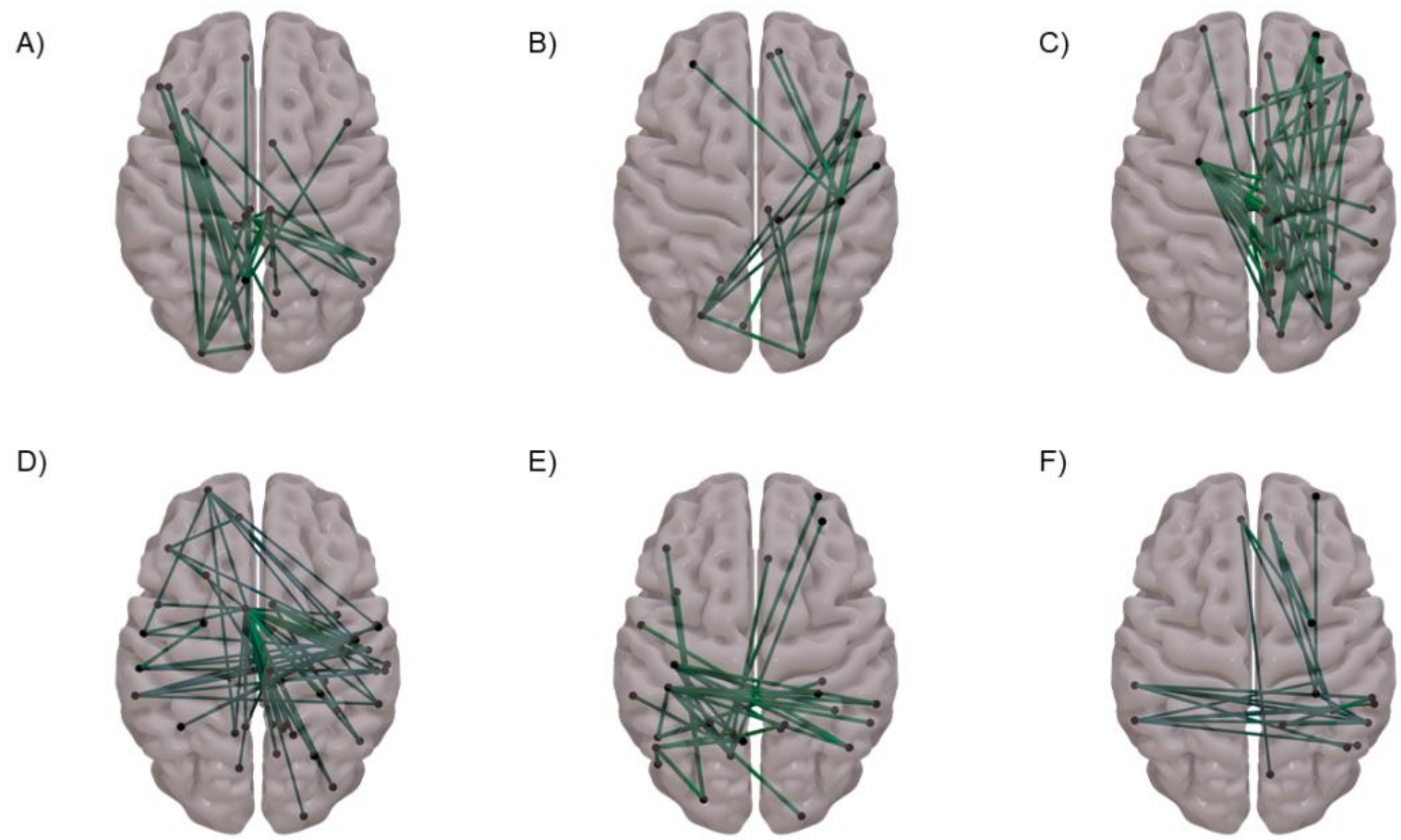


**Fig S2.** Connectivity modules in the theta-low alpha bands (4-9 Hz) computed through the clustering algorithm for the synchronous > asynchronous thresholding.

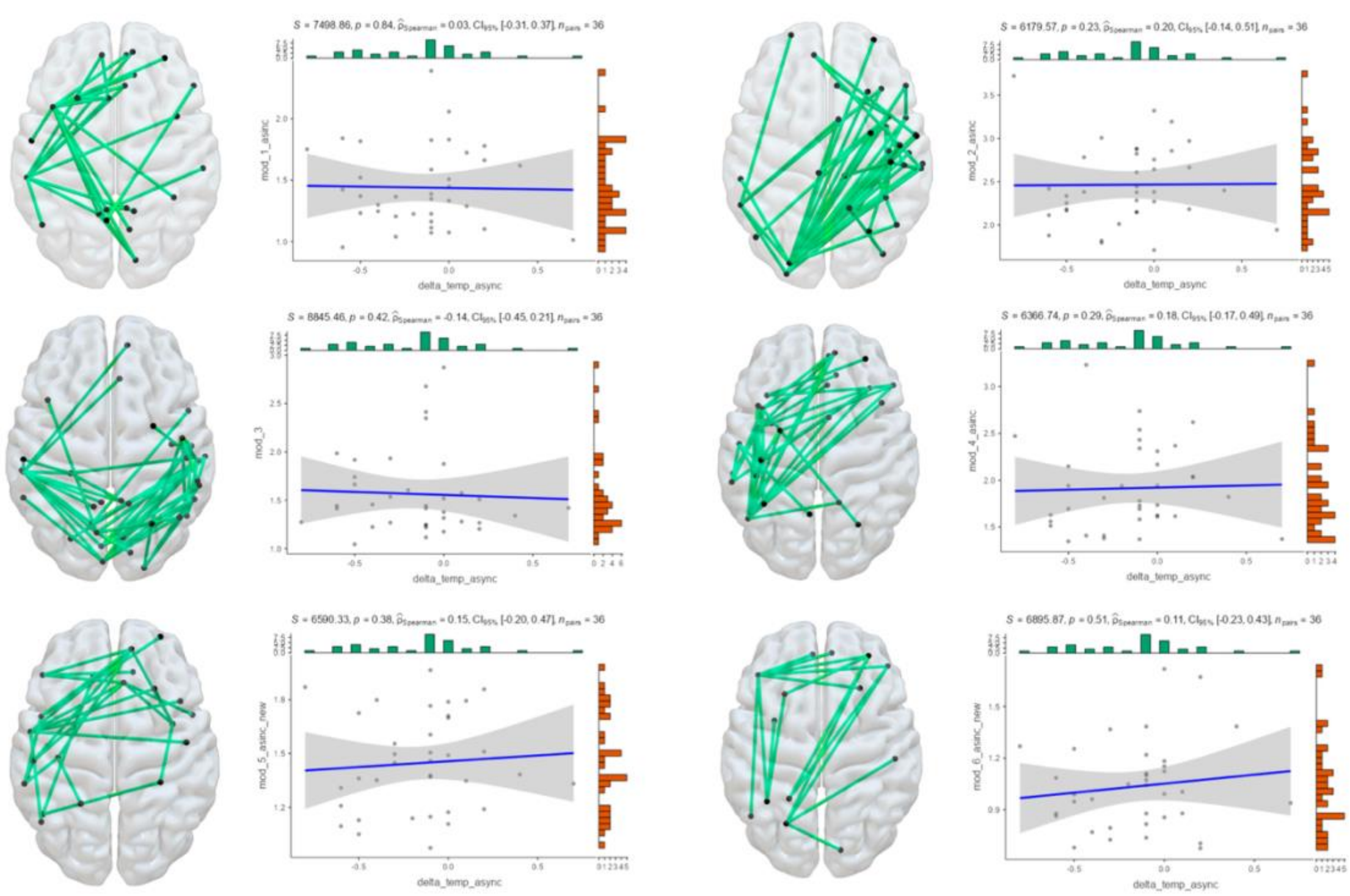

**Fig S3.** Connectivity modules in the theta-low alpha bands (4-9 Hz) computed through the clustering algorithm for the asynchronous > synchronous thresholding and the correlation between their network strength and temperature changes during the MBI.

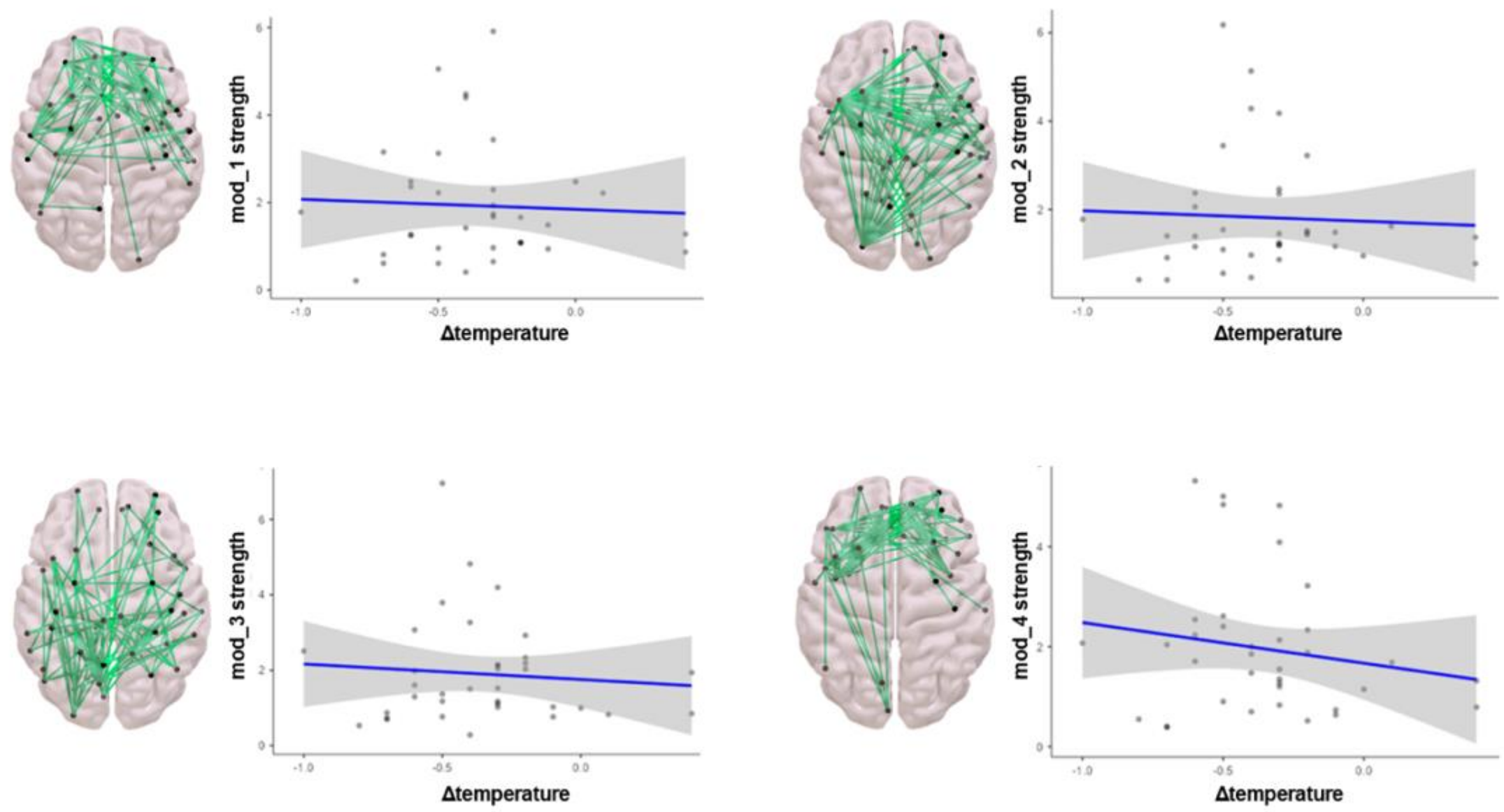


Fig S4. Connectivity modules in the beta band (13-30 Hz) and the correlation between their network strength and temperature changes during the MBI.
